# Genetic Mapping of *prod ^E.3.3^* , a New Lethal Allele of *prod*

**DOI:** 10.17912/micropub.biology.001236

**Published:** 2024-07-29

**Authors:** Emma Johnson, Talbot Kinney, Hannah Luellen, Rhiannan Amerud, Daysha R Anderson, Marie Anderson, Arnelyn Mae Andres, Rameel Arshad, Kylie Babin-Howard, Dede G Barrigah, Addison Beauregard, Leah Beise, Nolan Christofferson, Elijah L David, Luke DeWaard, Maya Diaz, Lily Donner, Natalie Ehlinger, Diellza Elmazi, Riley Engelhardt, Tamkanat Farheen, Mark M Figueroa, Soren Flaten, Madison Frush, Elizabeth Gonzalez, Jaylen Goolsby, Estefania Guzman, Logan Hanson, John Hejl, Jackson Heuschel, Brianna Higgins, Brylee Hoeppner, Daijah Hollins, Josette Knutson, Rachel Lemont, Mia Lopez, Samantha Martin, Trinity May, Abby McDade, Nearyroth Men, Ellie Meyer, Caroline R Mickle, Sebastian Mireles, Avery Mize, Jaiden Neuhaus, April Ost, Sarah Piane, Makenzie Pianovski, Aliya Rangel, Jessica Reyes, Alexandra Ruttenberg, Jacob D Sachs, Brandon Schluns, Nicholas Schroeder, Peighton R Skrobot, Cylie Smith, Sydney Stout, Andrew Valenzuela, Kaiden P Vinavich, Amber K Weaver, Michael Yager, Jose Zaragoza, Gabriela Zawadzki, Weam El Rahmany, Nicole L. Scheuermann, Hemin P Shah, Kayla L Bieser, Paula Croonquist, Olivier Devergne, Elizabeth E Taylor, Jacqueline K Wittke-Thompson, Jacob D Kagey, Stephanie Toering Peters

**Affiliations:** 1 Wartburg College, Waverly, Iowa, United States of America; 2 Anoka-Ramsey Community College, Coon Rapids, Minnesota, United States of America; 3 Northern Illinois University, DeKalb, Illinois, United States of America; 4 University of St. Francis, Joliet, Illinois, United States of America; 5 Nevada State University, Henderson, Nevada, United States of America; 6 University of Detroit Mercy, Detroit, Michigan, United States of America

## Abstract

The *E.3.3* mutation was generated in a Flp/FRT EMS screen for conditional mutations that cause growth and developmental defects in a genetic background that blocks apoptosis. The mutations were conditional, based on the *Dark ^82^* allele being present on the starting chromosome, and blocking canonical apoptosis in a homozygous state. The *E.3.3* mosaic eyes exhibit defects in eye development including patches of rough eye and irregular surface structure. Whole Genome Sequencing and complementation mapping revealed *E.3.3* as an allele of *prod* . Prod is a DNA-binding protein that binds satellite repeats and is involved in chromocenter formation during mitosis. Here we present a novel allele of *prod* , *prod ^E.3.3^* , that disrupts the functional region of the Prod protein resulting in disruption of typical eye structure, likely due to disruption of chromatid separation during development.

**Figure 1. Characterization of the prod E.3.3 allele in mosaic eyes and genetic sequencing.**
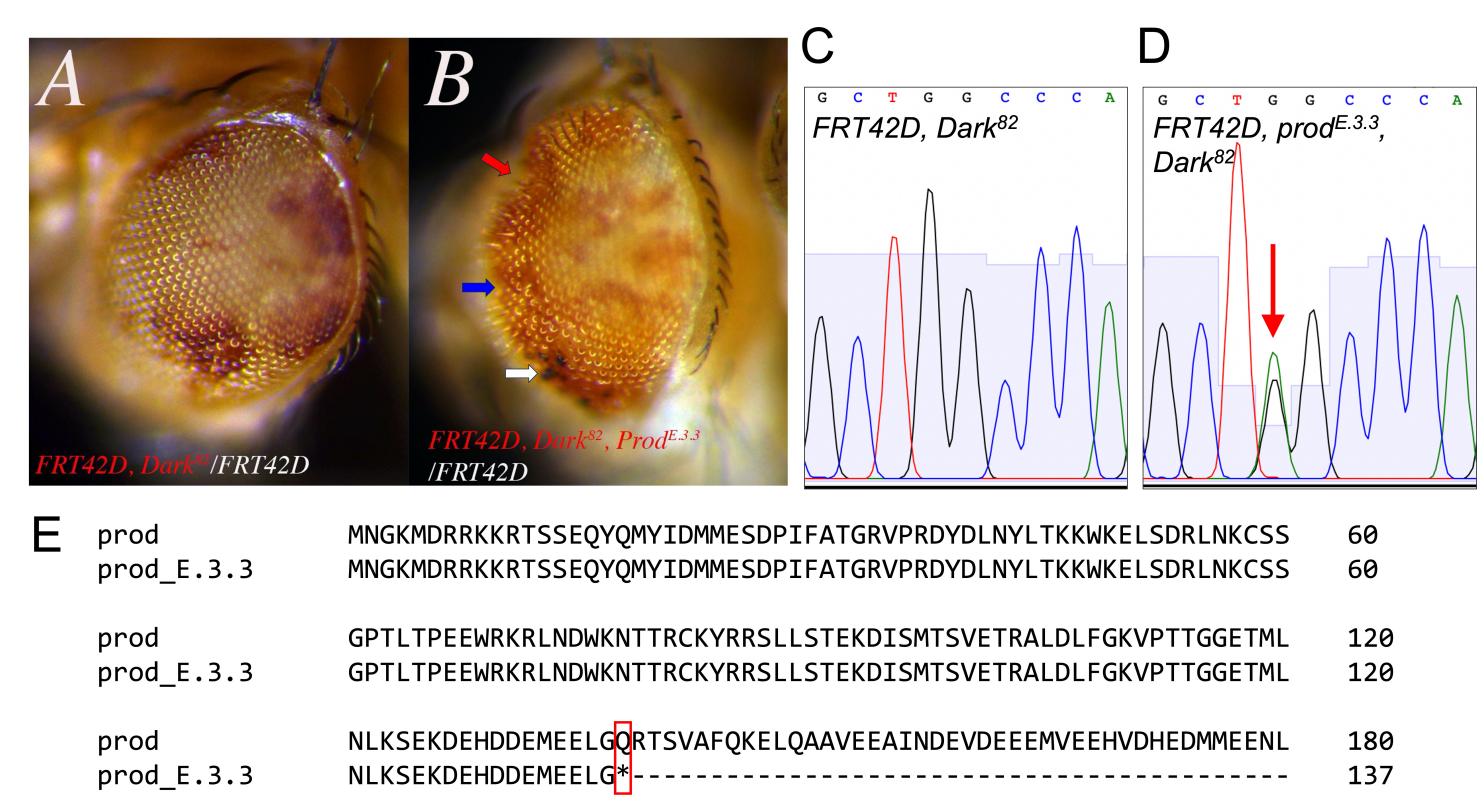
*
FRT42D, Dark ^82^* control mosaic eyes ( **A** ) have similar red (mutant cells): white (wild-type cells) ratio to *FRT42D, prod ^E.3.3^ , Dark ^82^* mosaic eyes ( **B** ). The *FRT42D, prod ^E.3.3^ , Dark ^82^* mosaic eyes are mis patterned (blue arrow), have potentially necrotic areas (white arrow), and have concave regions on the surface of the eye (red arrow). Sequencing of *prod* PCR products amplified from *FRT42D, Dark ^82^ /CyO* and *FRT42D, prod ^E.3.3^ , Dark ^82^ /CyO* heterozygous flies identified a heterozygous peak ( **D** , G→A, red arrow) present in the mutant flies that was not present in the control ( **C** ). This nucleotide change at position 2R:18970530 creates a premature stop codon in the protein at amino acid 138 ( **E** , Q→STOP, red box).

## Description

Undergraduate students in the Fly-CURE project (Merkle et al., 2023) characterize and map mutations identified in a screen for mutations that alter cell growth within the context of an undergraduate course. The mutant lines were generated in an EMS screen designed to identify genes that regulate cellular growth and development (Kagey et al., 2012) . The right arm of Chromosome 2 was screened using the Flp/FRT system to generate mitotic clones in a *Dark ^82^* background that suppresses apoptosis (Kagey et al., 2012) . The *Dark ^82^* loss-of-function mutation is the result of a transposon insertion that disrupts the *Dark* coding sequence, but also inserts the *mini-white ^+^* gene allowing the identification of homozygous mutant tissue in the eye based on color. The growth defects that result from the *E.3.3* mutation were examined by generating mitotic clones as a result of crossing *w ^-^ , ey>FLP; FRT42D* flies that express the FLP recombinase in the eye with flies that carry both the *E.3.3* allele and *Dark ^82^* allele on the *FRT42D* chromosome ( *w ^-^ ; FRT42D, Dark ^82^ , E.3.3/CyO* ). Tissue that was homozygous mutant for *E.3.3* and *Dark ^82^* was identified based on red color. These mosaic eyes were compared to the mosaic eyes from a control cross that did not include the *E.3.3* mutation ( *w ^-^ , ey>FLP; FRT42D* x *w ^-^ ; FRT42D, Dark ^82^ /CyO* ). The *E.3.3* mosaic eyes have a rough eye phenotype (blue arrow) with concave regions on the eye surface (red arrow) and some regions that appear glossy compared to the control mosaic eye (see Fig. 1A, B ). While the overall ratio of red to white tissue does not have a dramatic difference between the control and *E.3.3* , the overall patterning and organization of the eye has been disrupted and the overall eye size does appear a bit smaller, suggesting *E.3.3* has a role in eye development. Additionally, some mosaic *E.3.3* eyes also have black spots suggesting potential necrotic tissue in the mutant eye (white arrow).

In parallel, female *w ^-^ ; FRT42D, Dark ^82^ , E.3.3/CyO* flies were crossed with males from the Bloomington *Drosophila* Stock Center 2R Deficiency Kit in order to map the *E.3.3* mutation (Cook et al., 2012) . The Fly-CURE utilizes independent mapping experiments at each institution, so each cross was set up independently four times. For a cross to be designated a failure to complement, at least 50 curly winged flies have to be observed without any straight winged F1 progeny. These results were confirmed by independent replicates at each institution. Two of the 2R Deficiency Kit stocks failed to complement the *FRT42D, Dark ^82^ , E.3.3* chromosome: *Df(2R)ED2747* and *Df(2R)BSC331* . Both of these deficiencies include the *Dark* locus (Gramates et al., 2022) , so it is likely that the failure to complement is due to the lethal *Dark ^82^* allele. All of the remaining 2R Deficiency Kit stocks complemented the *E.3.3* mutation, indicating that either the *E.3.3* mutation lies in the same region of the chromosome as *Dark* , the *E.3.3* mutation is not lethal (and, thus, could not be identified through complementation testing) or the 2R Deficiency Kit used did not cover the region of the chromosome that contained the *E.3.3* . mutation. At this stage we took a similar approach to that of mutant *B.2.16* (Vrailas-Mortimer et al. 2021) . Whole Genome Sequence analysis of the *w ^-^ ; FRT42D, Dark ^82^ , E.3.3/CyO* genome identified a point mutation (G→A) in the *proliferation disrupter ( prod )* gene consistent with a premature stop codon (Vrailas-Mortimer et al. 2021) . The *prod* gene maps to nucleotides 18,969,404 to 18,971,133 on chromosome 2R ( *D. melanogaster* r6.56), which is located between two non-overlapping deficiencies in the Deficiency Kit, *Df(2R)ED3610* and *Df(2R)Exel6069* (Gramates et al., 2022) . The presence of this mutation in the *prod* gene and the location of *prod* in a region that is not covered by the Deficiency Kit used suggests that *E.3.3* was an allele of *prod , prod ^E.3.3^ .*

To confirm that *prod ^E.3.3^* is a lethal allele of *prod* , *w ^-^ ; FRT42D, Dark ^82^ , E.3.3/CyO* virgins were crossed with *prod ^E^* (hypomorphic) and *prod ^k08810^* (null)males that carry lethal alleles of the *prod* gene (Török et al., 1997; Gramates et al., 2022) . The *prod* alleles did not complement *prod ^E.3.3^* , supporting the hypothesis that the lethal hit in *E.3.3* is a null allele, *prod ^E.3.3^ .* Based on newly available data on Flybase, there are only three of the ten *prod* alleles that have been characterized based on the nature of the allele. *prod ^E.3.3^* is the fourth characterized *prod* allele (Öztürk-Çolak et al. (2024)).

To confirm the molecular nature of the *prod ^E.3.3^* mutation, we sequenced PCR products from *prod* in *w ^-^ ; FRT42D, Dark ^82^ , E.3.3/CyO* flies and *w ^-^ ; FRT42D, Dark ^82^ /CyO* flies. Three different sets of primers were designed to ensure that the products overlapped and covered the entire coding sequence of the *prod* gene. PCR products of the expected size were visualized by gel electrophoresis and purified products were sequenced (Eurofins Genomics). The sequence chromatograms for both the mutant and control strains were examined for overlapping peaks that indicated heterozygosity. Only one heterozygous site was present in the *E.3.3* sequences that was not present in the control sequences (see Fig. 1C, D ), and this site aligned with the mutation identified in the Whole Genome Sequence analysis. The sequenced mutation introduces a premature stop codon in the *prod* transcript ( Fig. 1E ) that interrupts the predicted coiled-coil domain of the protein, as well as terminates the protein sequence in a region that has been determined to be essential for DNA binding (Török et al., 2000). Thus, it is likely that the *prod ^E.3.3^* allele does not produce a functional Prod protein.

Based on both the genetic and molecular results, we conclude that *prod ^E.3.3^* is a newly identified allele of *prod .* The *prod* gene was initially identified as *l(2)88/10* in a P-element screen that identified lethal P insertions with overgrowth phenotypes (Török et al., 1993) . It was later demonstrated to play a role in chromatid separation during mitosis, with homozygous mutants exhibiting a decrease in mitotic index and cell death in highly proliferative cell types (Török et al., 1997) . The previously identified functions of *prod* are consistent with the identification of an allele in a mosaic screen for growth defects.

## Reagents

**Table:** 

Bloomington Stock Center 2R Deficiency Kit			
Deficiency Stocks	BDSC Stock #	Chromosomal Deletions	Complementation Results
*Df(2R)ED2747*	9278	2R:16829073..17097303	Failed to complement (overlaps *Dark ^82^* )
*Df(2R)BSC331*	24356	2R:16869330..17139923	Failed to complement (overlaps *Dark ^82^* )
Mutant alleles of individual genes			
Gene	BDSC Stock #	Allele	Complementation Results
*p* rod	42688	* prod ^E^*	Failed to complement
*prod*	10814	* prod ^k08810^*	Failed to complement

*w ^-^ ; FRT42D, Dark ^82^ /CyO* (Akdemir et al. 2006)

*w ^-^ ; FRT42D, Dark ^82^ , E.3.3/CyO* (this study)

*w ^-^ , ey>FLP; FRT42D* (BDSC 5616)

Bloomington Drosophila Stock Center 2R Deficiency Kit (Cook et al. 2012)

*y ^1^ w ^67c23^ ; P{w[+mC]=lacW} prod ^k08810^ /CyO* (BDSC 10814)

*y ^1^ w ^67c23^ ; prod ^E^ /CyO, y ^+^* (BDSC 42688)

*prod* forward primer 1: 5' CAT CGA GCA CGC AGG C 3'

*prod* reverse primer 1: 5' CTC CAT CTC GTC GTC GTG C 3'

*prod* forward primer 2: 5' GAT GCT CAA TCT GAA GTC CG 3'

*prod* reverse primer 2: 5' AGC TTA TTG CCG GAG GAG G 3'

*prod* forward primer 3: 5' ACG CCG TCG AGT ACG TCA C 3'

*prod* reverse primer 3: 5' CGA CTG CTT AGA CCC ACT GAT C 3'

## References

[R1] Akdemir F, Farkas R, Chen P, Juhasz G, Medved'ová L, Sass M, Wang L, Wang X, Chittaranjan S, Gorski SM, Rodriguez A, Abrams JM (2006). Autophagy occurs upstream or parallel to the apoptosome during histolytic cell death.. Development.

[R2] Cook RK, Christensen SJ, Deal JA, Coburn RA, Deal ME, Gresens JM, Kaufman TC, Cook KR (2012). The generation of chromosomal deletions to provide extensive coverage and subdivision of the Drosophila melanogaster genome.. Genome Biol.

[R3] Gramates LS, Agapite J, Attrill H, Calvi BR, Crosby MA, Dos Santos G, Goodman JL, Goutte-Gattat D, Jenkins VK, Kaufman T, Larkin A, Matthews BB, Millburn G, Strelets VB, the FlyBase Consortium. (2022). Fly Base: a guided tour of highlighted features.. Genetics.

[R4] Jagannathan M, Cummings R, Yamashita YM (2019). The modular mechanism of chromocenter formation in Drosophila.. Elife.

[R5] Kagey JD, Brown JA, Moberg KH (2012). Regulation of Yorkie activity in Drosophila imaginal discs by the Hedgehog receptor gene patched.. Mech Dev.

[R6] Merkle JA, Devergne O, Kelly SM, Croonquist PA, Evans CJ, Hwalek MA, Straub VL, Hamill DR, Peister A, Puthoff DP, Saville KJ, Siders JL, Villanueva Gonzalez ZJ, Wittke-Thompson JK, Bieser KL, Stamm J, Vrailas-Mortimer AD, Kagey JD (2023). Fly-CURE, a multi-institutional CURE using Drosophila, increases students' confidence, sense of belonging, and persistence in research.. J Microbiol Biol Educ.

[R7] Öztürk-Çolak A, Marygold SJ, Antonazzo G, Attrill H, Goutte-Gattat D, Jenkins VK, Matthews BB, Millburn G, Dos Santos G, Tabone CJ, FlyBase Consortium (2024). FlyBase: updates to the Drosophila genes and genomes database.. Genetics.

[R8] Török T, Tick G, Alvarado M, Kiss I (1993). P-lacW insertional mutagenesis on the second chromosome of Drosophila melanogaster: isolation of lethals with different overgrowth phenotypes.. Genetics.

[R9] Török T, Harvie PD, Buratovich M, Bryant PJ (1997). The product of proliferation disrupter is concentrated at centromeres and required for mitotic chromosome condensation and cell proliferation in Drosophila.. Genes Dev.

[R10] Török T, Gorjánácz M, Bryant PJ, Kiss I (2000). Prod is a novel DNA-binding protein that binds to the 1.686 g/cm(3) 10 bp satellite repeat of Drosophila melanogaster.. Nucleic Acids Res.

[R11] Vrailas-Mortimer AD, Aggarwal N, Ahmed NN, Alberts IM, Alhawasli M, Aljerdi IA, Allen BM, Alnajar AM, Anderson MA, Armstong R, Avery CC, Avila EJ, Baker TN, Basardeh S, Bates NA, Beidas FN, Bosler AC, Brewer DM, Buenaventura RS, Burrell NJ, Cabrera-Lopez AP, Cervantes-Gonzalez AB, Cezar RP, Coronel J, Croslyn C, Damery KR, Diaz-Alavez L, Dixit NP, Duarte DL, Emke AR, English K, Eshun AA, Esterly SR, Estrada AJ, Feng M, Freund MM, Garcia N, Ghotra CS, Ghyasi H, Hale CS, Hulsman L, Jamerson L, Jones AK, Kuczynski M, Lacey-Kennedy TN, Lee MJ, Mahjoub T, Mersinger MC, Muckerheide AD, Myers DW, Nielsen K, Nosowicz PJ, Nunez JA, Ortiz AC, Patel TT, Perry NN, Poser WSA, Puga DM, Quam C, Quintana-Lopez P, Rennerfeldt P, Reyes NM, Rines IG, Roberts C, Robinson DB, Rossa KM, Ruhlmann GJ, Schmidt J, Sherwood JR, Shonoda DH, Soellner H, Torrez JC, Velide M, Weinzapfel Z, Ward AC, Bieser KL, Merkle JA, Stamm JC, Tillett RL, Kagey JD (2021). *B.2.16*
is a non-lethal modifier of the
*
Dark
^82^
*
mosaic eye phenotype in
*Drosophila melanogaster*
.. MicroPubl Biol.

